# Interaction of Masitinib with Organic Cation Transporters

**DOI:** 10.3390/ijms232214189

**Published:** 2022-11-16

**Authors:** Saliha Harrach, Jasmin Haag, Martin Steinbüchel, Rita Schröter, Ute Neugebauer, Jessica Bertrand, Giuliano Ciarimboli

**Affiliations:** 1 Experimental Nephrology, Department of Internal Medicine D, University Hospital Münster, 48149 Münster, Germany; 2 Experimental Orthopaedics, University Orthopaedic Clinic, Medical Faculty, University Hospital Magdeburg, 39120 Magdeburg, Germany

**Keywords:** Masitinib, tyrosine kinase inhibitors, transport, organic cation transporter, repurposing

## Abstract

Tyrosine kinase inhibitors (TKI) such as Masitinib were reported to be useful as therapeutic options in malignant disorders and nonmalignant diseases, like coronavirus disease 2019 (COVID-19). Most kinases must be translocated into targeted cells by the action of specific transport proteins, as they are hydrophilic and not able to cross cell membranes freely. Accordingly, the efficacy of TKI in target cells is closely dependent on the expression of their transporters. Specifically, Masitinib is an organic cation and is expected to interact with organic cation transporters (OCT and Multidrug and Toxin Extrusion proteins—MATE-). The aim of this work was to characterize the interaction of Masitinib with different OCTs. Human embryonic kidney 293 cells stably transfected with murine or human OCT were used for the experiments. The interaction of Masitinib with OCTs was investigated using quenching experiments. The intracellular accumulation of this drug was quantified using high performance liquid chromatography. Our results identified interactions of Masitinib with almost all investigated mouse (m) and human (h) OCTs and hMATE1 and indicated OCT1 and hOCT2 to be especially potent Masitinib translocators across cell membranes. Interestingly, some important differences were observed for the interaction with murine and human OCTs. In the future, investigations concerning further in vitro and in vivo properties of Masitinib and its efficacy related to transporter-related uptake mechanisms under pathophysiological conditions should be performed. Clinical trials in humans and other animals with Masitinib have already shown promising results. However, further research is necessary to understand the disease specific transport mechanisms of Masitinib to contribute to a successful and responsible therapy employment.

## 1. Introduction

Tyrosine kinases (TKs) play an important role in biology and regulation of normal cell differentiation, survival, migration, and growth [1]. Aberrations of TK expression and activity can abnormally stimulate these processes and promote a constitutive activation of downstream TK signalling, resulting, for example, in the development of cancer [1,2]. Due to the devastating results of TK dysfunctions, much effort has been exerted in developing substances able to interfere with their signalling pathway, resulting in new therapeutic approaches based on monoclonal antibodies or small molecule TK inhibitors (TKIs) [3,4]. Focusing on small molecule TKIs, these inhibit abnormal TK signalling pathways by binding intracellular tyrosine kinases and blocking either the ATP-binding site or TK protein–protein interaction [5]. Several TKIs are used with success to treat cancers such as chronic myeloid leukaemia (CML), gastrointestinal stromal tumour, non-small cell lung cancer, and breast cancer [6]. Interestingly, TKIs have also been investigated as repurposed drugs for decreasing viral cellular production since TKIs modulate specific host functions that are used by many viruses, as for example the severe acute respiratory syndrome coronavirus-2 (Sars-CoV-2), the etiologic agent of coronavirus disease 2019 (COVID-19) for intracellular multiplication [7,8,9].

**Figure 1:**
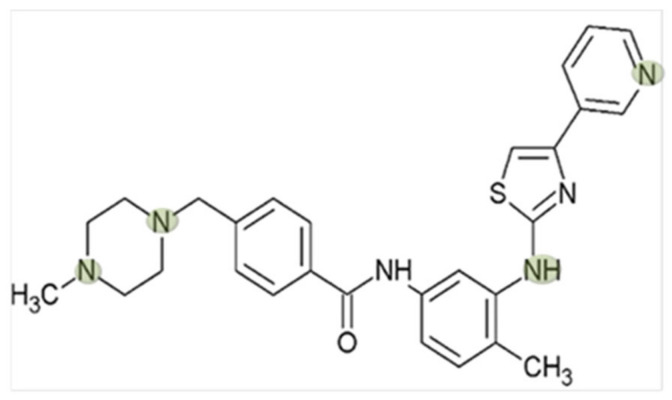
Structure of the TKI masitinib compiled using the ACD/ChemSketch program (Toronto, Canada). The marks (green) indicate predicted protonated sites in the molecule at pH 7.4.

In this context, a special interest has developed for Masitinib, a small molecule TKI (its structural formula is shown in Figure 1), which selectively and efficiently inactivates the kinase activity of the stem cell factor c-KIT, a receptor tyrosine kinase (RTK), of platelet-derived growth factor receptor α and β (PDGFRα/β), of cytoplasmic tyrosine kinase Lyn, and to a lesser extent, of fibroblast growth factor receptor 3 (FGFR3) [10]. Masitinib is approved for use in veterinary medicine, especially with dogs suffering from mast cell tumours (MCTs). About 20–30% of canine MCTs display mutations in the c-KIT gene, which promotes proliferation, differentiation, and degranulation of mast cells [11]. Additionally, this indirectly and negatively affects the controlling of proinflammatory and vasoactive mediator release [12]. Animal studies with dogs, rats, mice, and rabbits showed the good efficacy and acceptable toxicity of Masitinib, making it a candidate for patients’ treatment (see for example [3,13]). Several clinical trials with Masitinib have already displayed promising results in the treatment of inflammatory and neurological disorders, such as mastocytosis, rheumatoid arthritis, asthma, amyotrophic lateral sclerosis, and Alzheimer’s disease [14,15,16,17,18,19]. However, approvals of Masitinib for treatment of systemic mastocytosis and amyotrophic lateral sclerosis were refused since the efficacy and safety of Masitinib were not sufficiently demonstrated (European Medicines Agency, EMA, Assessment report EMA/641255/2017 and EMEA/H/C/4398). Nevertheless, a very recent randomized phase 3 clinical trial demonstrated that patients with primary or nonactive secondary progressive multiple sclerosis benefit from Masitinib treatment [20]. Moreover, it has been recently demonstrated that Masitinib is a potent competitive inhibitor of 3CLpro, the main SARS-CoV-2 protease [21], and Masitinib entered a phase 2 clinical trial (https://clinicaltrials.gov/ct2/show/record/NCT05047783) to test its antiviral efficacy in patients with symptomatic mild to moderate COVID-19.

Due to its protonated and hydrophilic character at physiological pH, Masitinib is an organic cation (Figure 1). Therefore, this molecule is not able to cross cell membranes freely but requires the help of transmembranous protein transporters. In this context, Masitinib might be a substrate/interaction candidate for members of the membrane transporter solute carrier 22A (SLC22A) and SLC47, which are translocators of organic cations (organic cation transporters, OCT, and Multidrug and Toxin Extrusion proteins, MATE, respectively) [22,23] and are known to mediate the cellular uptake of many drugs of cationic nature, such as metformin [24,25] and the ß-blockers atenolol [26] and nadolol [27]. Interestingly, several TKIs have been demonstrated to be OCTs or human MATE1 (hMATE1) substrates (e.g., saracatinib [28], imatinib [29,30], and pazopanib [31]) or to interact with OCT-mediated transport (e.g., sorafenib [32], imatinib, gefitinib, erlotinib and sunitinib [33]). OCT and MATE1 have a specific cellular expression: for example, they are essential for the secretion of organic cations (OCs) in the liver and kidney [34]. However, OCT organ distribution and their interaction with drugs can be species specific [35].

Therefore, in this work, the interaction of Masitinib with mouse and human OCTs and with hMATE1 was investigated.

## 2. Results

### 2.1. Inhibition of Uptake of the Fluorescent Organic Cation 4-(4-dimethylaminostyryl)-N-Methylpyridinium (ASP^+^) by Masitinib in Human Embryonic Kidney (HEK) 293 Cells Expressing Murine or Human OCT or hMATE1

The inhibition of ASP^+^ uptake measured using different concentrations of the TKI Masitinib was compared with ASP^+^ uptake without Masitinib (=100%) as control to determine the IC_50_ values (IC_50_ = Masitinib concentration, which inhibits 50% of ASP^+^ uptake) in HEK293 cells transfected with murine (m) or human (h) OCT. Moreover, since OCTs mediate the cellular accumulation of OCs in hepatocytes (hOCT1) and renal proximal tubules cells (hOCT2) and hMATE1 the excretion of OCs into the bile and urine [36,37], we studied Masitinib interaction with ASP^+^ uptake mediated by hMATE1. hMATE1 is an important member of the vectorial hepatic and renal excretion system of OCs since it mediates the last step of this process.

**Figure 2:**
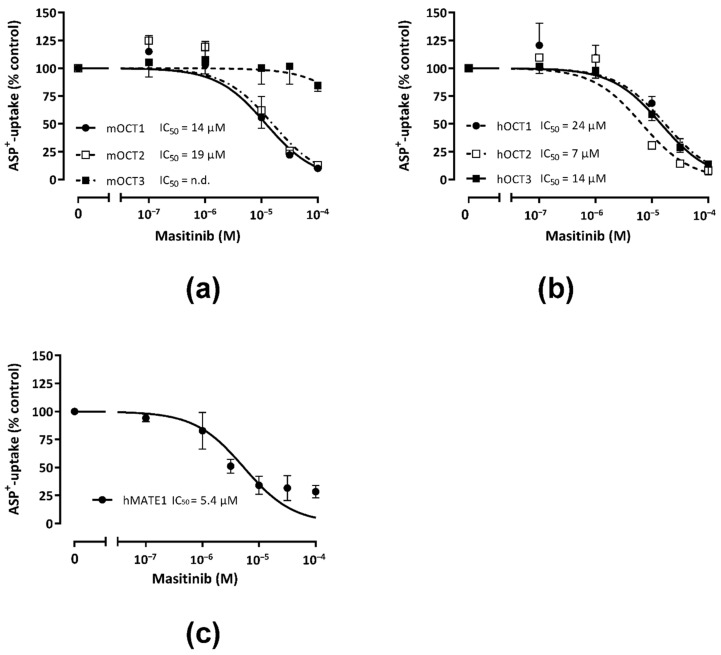
The graphs show the concentration-dependent inhibition of the initial ASP^+^ uptake by Masitinib in HEK293 cells stably expressing murine (panel (**a**)), or human (panel (**b**)) OCTs and human MATE1 (panel (**c**)). The *y*-axis displays the ASP^+^-uptake in % of control experiments without Masitinib (=100%). Values are mean ± SEM calculated in 18–50 replicates measured in at least 3 independent experiments. The IC_50_-values for each OCT are indicated in the inserts. Panel a shows the inhibition of ASP^+^ (1 µM) uptake by increasing Masitinib concentrations in HEK293 cells stably transfected with mOCT1- (closed dots), mOCT2- (open squares), or mOCT3 (closed squares). For mOCT3, the IC_50_-value could not be determined (n.d.), while Masitinib inhibited the ASP^+^ uptake by mOCT1 and mOCT2 with a similar apparent affinity (14 and 19 µM, respectively). Panel b shows the inhibition of ASP^+^ (1 µM) uptake by increasing Masitinib concentrations in HEK293 cells stably transfected with hOCT1- (closed dots), hOCT2- (open squares), or hOCT3 (closed squares). Masitinib inhibited the ASP^+^ uptake by hOCT1, hOCT2, and hOCT3 with a similar apparent affinity (24, 7, and 14 µM, respectively). Panel c shows the inhibition of ASP^+^ (1 µM) uptake by increasing Masitinib concentrations in HEK293 cells stably transfected with hMATE1- (closed dots). Masitinib inhibited the ASP^+^ uptake by hMATE1 with a high apparent affinity (5 µM).

The IC_50_-values do not represent a direct measurement of the transporter affinity for the competing substance and therefore are referred to as “apparent affinity”. Figure 2 shows the results of the concentration-dependent inhibition of ASP^+^ uptake by Masitinib in HEK293 cells stably expressing murine (m) (panel a) or human (h) (panel b) OCT-1, -2, or -3, or hMATE1 (panel c). The IC_50_ values for the Masitinib inhibition of ASP^+^ transport mediated by h and m OCT paralogs and orthologs and hMATE1 are summarized in Table 1. IC_50_-values of mOCT1 (14 µM, *n* = 20–50) and mOCT2 (19 µM, *n* = 23–43) lie below 20 µM, indicating a similar apparent affinity for Masitinib. In contrast, Masitinib seemed to not to interact with ASP^+^ transport mediated by mOCT3. Masitinib inhibited the ASP^+^ uptake in a concentration-dependent manner by hOCT1, hOCT2, hOCT3, and hMATE1. Interestingly, low Masitinib concentrations from 10^−7^–10^−6^ M caused a slight stimulation of the ASP^+^ uptake in all three OCT subtypes. hOCT2-transfected HEK293 cells showed a higher apparent affinity for Masitinib (IC_50_-value: 7 µM, *n* = 19–39) compared with hOCT1 (IC_50_-value: 24 µM, *n* = 20–42) and hOCT3 (IC_50_-value: 14 µM, *n* = 20–44). Masitinib also inhibited ASP^+^ transport mediated by hMATE1 with a high apparent affinity (IC_50_-value: 5 µM, *n* = 18–37). Based on these results, we could show that Masitinib is able to interact with the ASP^+^ uptake via mOCT1-2, hOCT1-3, and hMATE1, suggesting that they are potential Masitinib transporters.

### 2.2. Accumulation of Masitinib Mediated by Murine and Human OCTs Stably Expressed in HEK293 Cells

**Figure 3:**
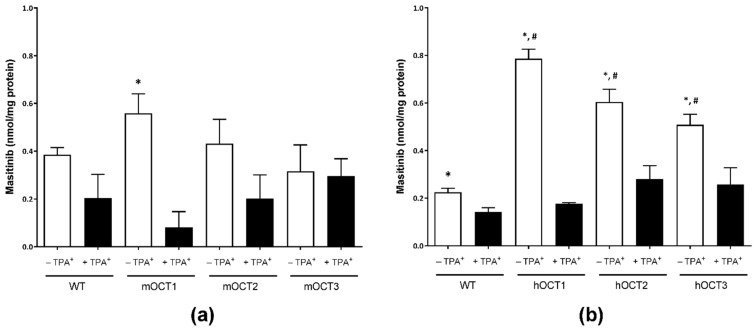
Columns indicate Masitinib accumulation in WT- (*n* = 12) and m (panel (**a**)) or h (panel (**b**)) OCT1-3 stably expressing HEK293 cells in the presence (+ TPA^+^) or absence (− TPA^+^) of 1 mM of TPA^+^. Columns show the mean ± SEM of Masitinib uptake given as nmol Masitinib/mg protein measured in 9–12 replicates in 3 independent experiments. In panel a, * shows a statistically significant difference to experiments performed with mOCT1-expressing cells in the presence of 1 mM of TPA^+^ (unpaired *t*-test). In panel b, * shows a statistically significant difference to respective experiments performed in the presence of 1 mM of TPA^+^ (unpaired *t*-test), while # shows a statistically significant difference to WT without TPA^+^ (ANOVA with Tukey post-test).

The interaction of Masitinib with the ASP^+^ uptake mediated by OCT and hMATE1 does not necessarily predict whether Masitinib is also a substrate of these transporters. Therefore, to answer this question, we measured the intracellular accumulation of Masitinib in HEK 293 cells stably expressing mouse or human OCTs and hMATE1 by means of HPLC determination. To estimate the non-OCT-mediated Masitinib transport, cellular accumulation in the presence of a high concentration (1 mM) tetrapentylammonium (TPA^+^), a specific OCT1-3 inhibitor, was also measured. Figure 3 summarizes the results of accumulation experiments with murine (panel a) and human (panel b) OCTs, respectively.

Figure 3a shows that slight specific Masitinib uptake was also present in WT-HEK293 cells (all data are given as mean Masitinib amount in nmol/mg protein ± SEM; 0.38 ± 0.03 nmol/mg, *n* = 9). The cells transfected with mouse transporters showed the following Masitinib accumulation: (a) mOCT1-HEK293, 0.56 ± 0.08 nmol/mg protein; (b) mOCT2-HEK293, 0.43 ± 0.10 nmol/mg protein; and (c) mOCT3-HEK293, 0.32 ± 0.11 nmol/mg protein. The Masitinib transport in the presence of TPA^+^ was lower in every cell line than in experiments without TPA^+^ (0.20 ± 0.10, 0.08 ± 0.07, 0.20 ± 0.09, and 0.29 ± 0.07 nmol Masitinib/mg protein for the WT-, mOCT1-, mOCT2-, and mOCT3-HEK293 cells, respectively), but this difference reached a statistically significant difference only in mOCT1-HEK293 cells (unpaired *t*-test).

Figure 3b shows again that slight specific Masitinib uptake was present in WT-HEK293 cells (0.23 ± 0.02 nmol/mg, *n* = 9). The cells transfected with human transporters showed the following Masitinib accumulation: (a) hOCT1-HEK293, 0.79 ± 0.04 nmol/mg protein; (b) hOCT2-HEK293, 0.60 ± 0.05 nmol/mg protein; and (c) hOCT3-HEK293, 0.51 ± 0.05 nmol/mg protein. hOCT1-3-expressing cells accumulated significantly more Masitinib than did cells transfected with the empty vector (WT-HEK293 cells, ANOVA with Tukey post-test). The Masitinib transport in the presence of TPA^+^ was significantly lower in every cell line than in those without TPA^+^ (unpaired *t*-test) and not different between the different cell lines (0.14 ± 0.02, 0.18 ± 0.01, 0.28 ± 0.06, and 0.26 ± 0.07 nmol Masitinib/mg protein for WT-, hOCT1-, hOCT2-, and hOCT3-HEK293 cells, respectively; ANOVA with Tukey post-test).

**Figure 4:**
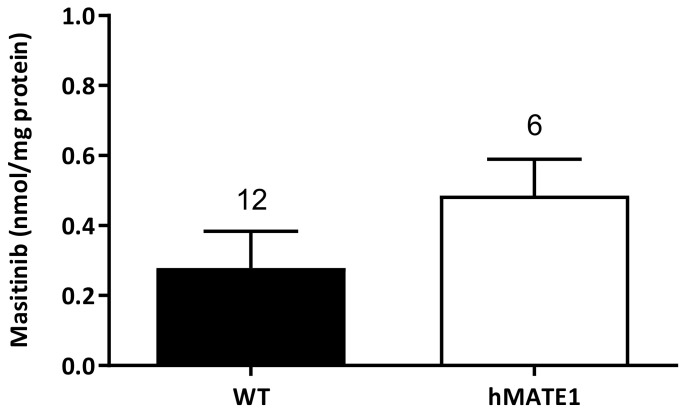
Columns indicate Masitinib temperature-dependent accumulation (accumulation measured at 37 °C minus accumulation measured at 4 °C) in WT- (*n* = 12) and hMATE1- (*n* = 6) HEK293 cells. Columns show the mean ± SEM of Masitinib uptake given as nmol Masitinib/mg protein measured in 12 and 6 replicates for WT- and hMATE1-HEK293 cells, respectively, in 3 independent experiments.

Figure 4 shows the Masitinib uptake in WT-HEK293 cells (0.28 ± 0.1 nmol/mg, *n* = 12) and in HEK293 cells stably expressing hMATE1 (0.49 ± 0.1 nmol/mg, *n* = 6). The accumulation measured at 4 °C was subtracted from that measured at 37 °C to calculate the temperature-dependent Masitinib accumulation in these cells.

### 2.3. Saturation Kinetics

**Figure 5:**
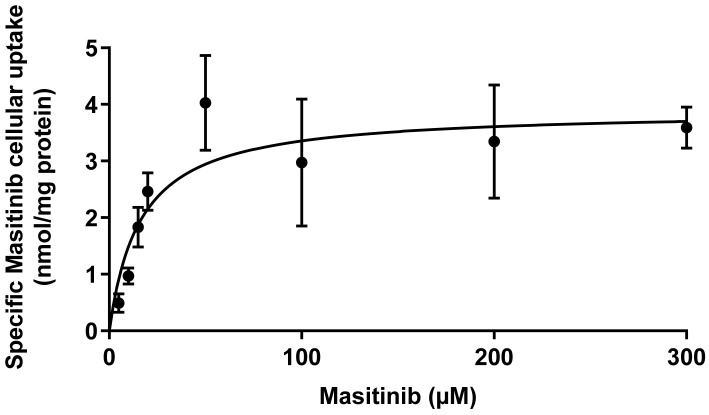
Saturation kinetic of Masitinib in hOCT1-HEK293 cells. Three independent experiments were performed. The values are shown as mean ± SEM. Fitting of the curve according to a Michaelis–Menten kinetic gave the following values for K_m_ and V_max_:K_m_ = 16.3 ± 5.2 µM Masitinib and V_max_ = 3.9 ± 0.3 nmol Masitinib/mg protein (*n* = 3).

Since hOCT1 mediated the highest cellular Masitinib accumulation, the kinetic characteristics of this transport were evaluated measuring Masitinib accumulation in hOCT1-HEK293 cells dependent on the Masitinib concentration. To do this, cells were incubated for 10 min with 1*–*300 µM Masitinib, and then the Masitinib quantity/mg protein was measured in cell lysates with the HPLC method. Unspecific accumulation was determined in the presence of 1 mM of TPA^+^ and subtracted from the total Masitinib uptake. The determined saturation curve is shown in Figure 5. The resulting maximal turnover velocity (V_max_) and the Michaelis–Menten constant (K_m_) were 3.9 ± 0.3 nmol Masitinib/mg protein and 16.3 ± 5.2 µM Masitinib, respectively.

## 3. Discussion

The aim of this work was to study the interaction of the TKI Masitinib with OCTs and hMATE1 in vitro, examining its inhibitory potential and the apparent transporter affinity to identify the main transporter system mediating its transport into the target cells.

To exert its biological action, Masitinib requires that it is transported across cell membranes since at physiological pH, it is an organic cation with a molecular mass of 498.64 g/mol that cannot pass freely through the cell membrane by diffusion. For this reason, the cellular uptake of Masitinib might be mediated by polyspecific organic cation transporters of the SLC22A family (OCT1-3) and SLC47 family (MATE) [38,39].

We investigated and compared the apparent affinities for Masitinib to OCT and hMATE1 by measuring the Masitinib-induced inhibition of ASP^+^ uptake since ASP^+^ is a substrate for OCT1-3 and hMATE1 [29,33]. We found that Masitinib efficiently inhibits the ASP^+^ uptake by most of these transporters with IC_50_-values in the micromolar range, except for mOCT3. Among the murine transporters, mOCT1 showed the strongest interaction with Masitinib, followed by mOCT2. Among the human transporters, hMATE1 and hOCT2 showed the strongest apparent affinity for Masitinib, closely followed by hOCT3 and hOCT1.

The comparison between the respective murine and human transporters indicates higher similar apparent affinities for Masitinib in OCT1 and OCT2 than in OCT3 (summarized in Table 1). However, murine and human OCT3 interact with Masitinib remarkably differently. mOCT3 showed the weakest interaction with Masitinib. These results could be useful for the interpretation of the translational relevance of studies performed in mouse models. Masitinib was able to inhibit the ASP^+^ uptake mediated by hMATE1 with a great apparent affinity (5 µM).

Interestingly, we observed that low concentrations of Masitinib between 10^−7^–10^−6^ M enhanced the ASP^+^ uptake by some transporters, which was followed by an inhibition at higher Masitinib concentrations. Others studying the interaction of other substances such as 1-methyl-4-phenylpyridinium (MPP^+^) with rOCT3 have already observed this activating effect [40]. A theory about the mechanisms behind this phenomenon suggests the existence of distinct but overlapping binding sites for ligand-binding and translocation processes, so called high-affinity and low-affinity binding sites. According to this model, Masitinib would induce an allosteric transition at low concentrations, preferring the interaction and/or transport of ASP^+^ at the high affinity binding site, while higher Masitinib concentrations would cause a direct blocking of the low-affinity site resulting in ASP^+^ uptake inhibition [41].

However, the ASP^+^ inhibition model does not directly prove whether Masitinib is an actual substrate for OCTs or hMATE1 and whether it is intracellularly accumulated. To determine whether Masitinib is taken up into the cells by OCTs, the accumulation of Masitinib into these cells was studied at 37 °C. Accordingly, the quantification of intracellular Masitinib via HPLC showed significantly higher cellular Masitinib concentrations at 37 °C compared to 4 °C indicating a temperature-dependent uptake of Masitinib (not shown). Interestingly, we also observed temperature-dependent Masitinib uptake in WT-HEK293 (not shown). Endogenously expressed transporters might mediate this uptake. Possible candidates may be other members of the SLC transporter family, such as hOATP1A2 und hOATP1B3, which were also found to transport the analogue TKI imatinib [42].

Our results reveal that compared with WT-KEK293 cells, hOCT1-, hOCT2-, hOCT3-, and mOCT1-expressing HEK293 cells are able to translocate significantly more Masitinib, indicating a higher substrate specificity of Masitinib for these transporters. The overexpression of hMATE1 did not significantly increase Masitinib cellular accumulation, showing that even though Masitinib inhibits ASP^+^ uptake mediated by hMATE1, it is not a substrate of this transporter. The Masitinib uptake mediated by hOCT1, hOCT2, hOCT3, and mOCT1 could be inhibited by co-incubation with the organic cation TPA^+^, showing the specificity of the transport. Interestingly, Masitinib transport observed in WT cells was also inhibited by TPA^+^, suggesting the contribution of endogenously expressed TPA^+^ inhibitable transporters to Masitinib uptake.

Focusing on hOCT1, we determined its kinetic properties, which are important for a direct comparison of Masitinib transport efficiency by the different transporters under study. In vitro, Masitinib showed a K_m_-value of 16 µM, which was in accordance with the measured IC_50_ for hOCT1 (24 µM). 

TKIs such as Masitinib have been reported to be useful as therapeutic options in malignant disorders, including CML and nonmalignant diseases, such as rheumatoid arthritis [43,44]. Most kinase inhibitors must be translocated into targeted cells by the action of specific transport proteins, as they are hydrophilic and not able to cross cell membranes freely. Accordingly, the efficacy of TKIs in target cells is closely dependent on the expression of such transporters.

Our results identified interactions of Masitinib with almost all investigated OCTs and indicated hOCT1 in particular to be a potent Masitinib translocator across the cell membrane. Therefore, it can be suggested that Masitinib-based therapy will be especially effective in target cells expressing hOCT1. Depending on the administered dose, the maximum (or peak) serum concentration C_max_ of Masitinib has been reported to reach values in the micromolar range (European Medicines Agency, EMA, Assessment report EMA/641255/2017), suggesting an importance of the interaction with OCT for cellular Masitinib uptake under pharmacological treatment. Interestingly, Masitinib is a promising agent for inhibition of SARS-CoV-2 replication in infected cells. Since both OCT1 and OCT3 are expressed in human nasal epithelial cells [45,46] and in different types of lung cells [47], the use of Masitinib may be effective in blocking viral production in airway cells.

Nearly all TKIs are orally administered drugs, and for this reason, expression of OCTs (OCT1) in enterocytes may be important for drug adsorption [48]. OCTs in the liver and in the kidneys mediate the first step of OC hepatic and renal excretion: that is, the uptake in hepatocytes and renal proximal tubules cells by OCT1 and OCT2, respectively. The final step of this excretion process is OC secretion into the bile and into the urine, which is mediated by transporters such as hMATE1. Therefore, the results suggest that Masitinib enters hepatocytes and renal proximal tubules cells by hOCT1 and hOCT2, respectively, but it is not efficiently excreted into the bile or into the urine since it is not a good substrate for hMATE1. In a dose-escalation study conducted in patients with advanced and/or metastatic cancer, it was found that during Masitinib treatment, 27.5% of the patients showed signs of hepatobiliary disorders and 22.5% showed signs of renal disorders [49]. These toxic effects of Masitinib may be related to its poor excretion by hMATE1.

In the future, further investigations concerning the in vitro and in vivo properties of Masitinib and its efficacy related to transporter-related uptake regulation under pathophysiological conditions should be performed. Clinical trials in humans and animals with Masitinib have already shown promising results. However, further research is necessary to understand the transport mechanisms of Masitinib to contribute successful and reliable therapeutic application.

## 4. Materials and Methods

### 4.1. Cell Culture

Human embryonic kidney (HEK) 293 cells stably expressing hOCT1-3, hMATE1, or mOCT1-3 or the respective empty vector (these cell lines were indicated as wildtype, WT- cells) were used for the experiments. Generation of these cell lines has been described elsewhere [50,51,52,53]. Characterization of transporter expression in these cells is reported in the Supplementary Materials (Supplementary Materials Tables S1 and S2, Supplementary Materials Figures S1–S6). HEK293 cells were maintained at 37 °C in 5% CO_2_ and 50 mL cell culture flasks (Greiner, Frickenhausen, Germany). Cell medium consisted of Dulbecco’s minimal Eagle’s medium (Biochrom, Berlin, Germany) supplemented with 10% fetal bovine serum, 1 g/L of glucose, 2 mM of glutamine, 3.7 g/L of NaHCO_3_, and 100 U/mL of streptomycin/penicillin (Biochrom). Selection of cells transfected with OCTs was assured by the addition of 0.8 mg/mL of Geneticin (PAA Laboratories) or, for hMATE1-HEK293 cells, by the addition of 0.5 mg/mL of hygromycin B (Invitrogen). Cell cultures were grown on 96-, 24-, or 12-well plates until 80–90% confluence was reached. Experiments were performed with cells from passages 20–65.

### 4.2. Fluorescence Measurements

**Figure 6:**
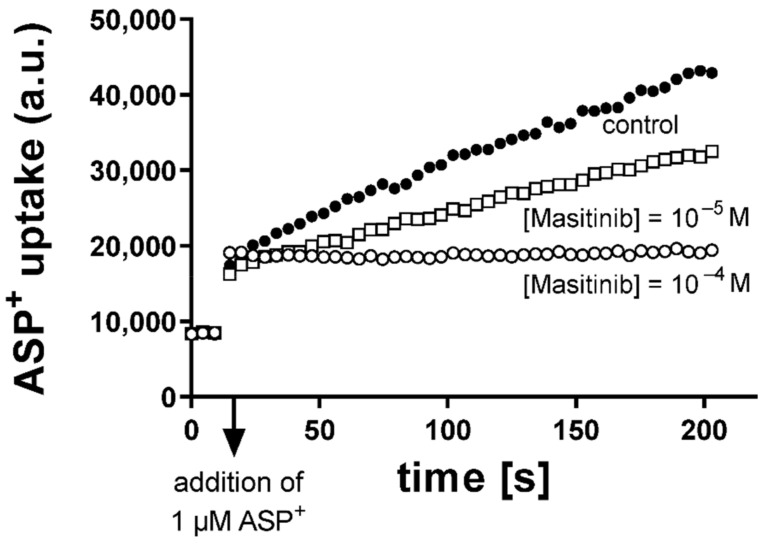
Example of experiments, where the uptake of 1 µM ASP^+^ (in arbitrary units, a.u.) over time by hOCT3-transfected HEK293 cells was measured. The uptake of ASP^+^ alone (control, closed dots) or in the presence of Masitinib at concentrations of 10^−5^ M (open squares) or 10^−4^ M (open dots) over time is shown. A concentration of 10^−4^ M Masitinib inhibited ASP^+^ uptake almost completely. The arrow shows the time point of ASP^+^ addition.

The activity of OCT and hMATE1 was evaluated measuring the uptake of fluorescent organic cation 4-(4-dimethylaminostyryl)-N-methylpyridinium (ASP^+^) at a concentration of 1 µM, as described elsewhere [29,54]. Measurements were performed using a microplate fluorescence reader with excitation at 465 nm and emission at 590 nm (Infinite F200, Tecan, Switzerland). This method allowed us to dynamically measure the transporter activity with a high time resolution (5 s). HEK293 cells expressing OCT or the empty vector (WT) were seeded into 96-well plates and grown to 80–100% confluence. Before measurements, cells monolayers were washed with Ringer-like solution containing (in mM) NaCl 145, K_2_HPO_4_ 1.6, KH_2_PO_4_ 0.4, D-glucose 5, MgCl_2_ 1, and calcium gluconate 1.3, with the pH adjusted to 7.4 at 37 °C. The apparent affinities (IC_50_ = concentration, which inhibits 50% of ASP^+^ uptake) of the transporters for Masitinib were evaluated using cis-inhibition of ASP^+^ uptake by Masitinib (10^−7^ to 10^−4^ M), as described previously [55]. Figure 6 shows an example of the experiments, where uptake of 1 µM of ASP^+^ has been measured in the presence and absence of Masitinib. Masitinib (LC Laboratories, Woburn, MA, USA) was diluted from a 5 mM stock in DMSO to the desired concentration with Ringer-like solution. ASP^+^ uptake without Masitinib was set to 100% (control). Slopes of fluorescence increase were linearly fitted and used as ASP^+^ uptake measure.

### 4.3. Measurement of Masitinib in Cell Lysates by High Performance Liquid Chromatography (HPLC)

Since the interaction between Masitinib and ASP^+^ cannot indicate whether Masitinib is also transported into the cells by a specific transporter, the transport of Masitinib by OCTs and hMATE1 was quantified using reversed-phase HPLC. The mobile phase consisted of (A) 0.1% formic acid and (B) acetonitrile and was delivered at 150 µL/min in a gradient mode. After delivery of 100% A for 1 min, a linear gradient to 50% B was applied and maintained for 10 min. Thereafter isocratic elution with 100% B was applied for 5 min, and then the column was re-equilibrated with 100% A for at least 5 min (Table 2). Masitinib, detected by UV absorption at 254 nm, has a retention time of 8.6 min. An example of the chromatograms obtained by HPLC is presented in Supplementary Materials Figure S7.

The chromatographic system consisted of an Accela 600 Pump, an automatic sampler, an UV detector set to 254 nm for detection, and a degasser (Thermo Fisher Scientific, Waltham, MA, USA). Separation was carried out on an Accucore C18 column (Thermo Fisher Scientific) equipped with a C18 guard column (Phenomenex, Aschaffenburg, Germany). The data were acquired by ChromQuest 5.0 chromatography software (Thermo Fisher Scientific).

### 4.4. Preparation of Samples for Detection of Cellular Masitinib Accumulation by HPLC

For these experiments, confluent cells growing in 12-well plates were used. In these cells, temperature-dependent Masitinib accumulation in the presence and absence of specific transporter inhibitors was investigated. To do this, cells stably expressing hOCT1-3, hMATE1, or mOCT1-3 or WT cells (transfected with empty vector, control experiments) were incubated with 300 µL of 10 µM Masitinib at 37 °C and also under inhibition of metabolic processes at 4 °C. After the incubation, each well was washed with 300 µL of ice-cold Ringer-like solution to stop the transport activity. As a last step, the cells were lysed with 200 µL of 0.1% formic acid, resuspended with the help of a cell scraper, and filled into sterile 1.5 mL tubes. The lysates were placed into an ultrasonic bath for 10 min and then centrifuged at 2000× *g* for 10 min at 4 °C. The supernatant was collected and used for quantification of Masitinib by HPLC and of proteins by picodrop in triplicate (Picodrop Ltd., Hinxton, UK). For saturation kinetic experiments, hOCT1-HEK293 cells growing in 24-well plates were used. Cells were incubated for 10 min with 150 µL of different Masitinib concentrations, washed with 200 µL ice-cold Ringer-like solution, and finally lysed with 150 µL of 0.1% formic acid. Masitinib concentration in cell lysates was measured by HPLC as described above. The measured Masitinib concentration in cell lysates was normalized to the protein concentration in the sample.

### 4.5. Statistical Analysis

Data were analysed using GraphPad Prism version 9.0 (GraphPad Software, Inc., San Diego, CA, USA). Data presented in this work are expressed as mean values ± SEM, with *n* referring to the number of replicates and N referring to the number of independent experiments. IC_50_-, K_m_-, and V_max_-values were obtained by a nonlinear sigmoidal concentration response curve fitting. When indicated, unpaired *t*-test and ANOVA test with Tukey post were applied to prove statistical significance (*p* < 0.05).

## Figures and Tables

**Table 1:** Summary of IC_50_-values for Masitinib inhibition of ASP^+^ transport mediated by murine and human OCT paralogs and orthologs and hMATE1.

	IC_50_ (µM) (logIC_50_ ± SEM) for Masitinib and Number of Replicates (n) Measured in 3 Independent Experiments	
Transporters	Murine	Human
OCT1	14 (−4.8 ± 0.3) *n* = 20–50	24 (−4.6 ± 0.2) *n* = 19–39
OCT2	19 (−4.7 ± 0.3) *n* = 23–43	7 (−5.1 ± 0.1) *n* = 20–42
OCT3	n.d. ^1^ *n* = 19–31	14 (−4.8 ± 0.1) *n* = 20–42
MATE1		5 (−5.3 ± 0.3) *n* = 18–37

^1^ n.d. = in this case the IC_50_ value could not be determined.

**Table 2:** Gradient program for HPLC measurements.

Time (min)	A % (0.1% Formic Acid)	B % (Acetonitrile)	Flow Velocity (µL/min)
0	100	0	150
1	100	0	150
10	50	50	150
11	0	100	150
15	100	0	150
20	100	0	150

## Data Availability

Not applicable.

## Supplementary Materials

The following supporting information can be downloaded at: https://www.mdpi.com/article/10.3390/ijms232214189/s1.
