# Supplementary material for: Interaction of Masitinib with Organic Cation Transporters
Source: Int J Mol Sci. 2022 Nov 16;23(22):14189. doi: 10.3390/ijms232214189 (PMC9693006; doi:10.3390/ijms232214189)
Supplement: Supplementary file 1 [file ijms-23-14189-s001.zip › ijms-1993853-supplementary.pdf]

# Supplementary Material

# Interaction of Masitinib with Organic Cation Transporters

**Saliha Harrach [1], Jasmin Haag [1], Martin Steinbüchel [1], Rita Schröter [1], Ute Neugebauer [1], Jessica Bertrand [2] and Giuliano Ciarimboli [1,*]**

[1] Experimental Nephrology, Department of Internal Medicine D, University Hospital Münster, 48149 Münster, Germany
[2] Experimental Orthopaedics, University Orthopaedic Clinic, Medical Faculty, University Hospital Magdeburg, 39120 Magdeburg, Germany
* Correspondence: gciari@uni-muenster.de; Tel.: +49-251-83-56981

Characterization of OCT-mRNA expression by PCR-analysis of the cells used in this study

To evaluate the efficacy of genetic manipulation and at the same time the presence of endogenously expressed transporters, the expression of OCT in the cell lines was evaluated by reverse transcriptase PCR (RT-PCR) and, for human OCT, also by real time PCR analysis, which allows the quantitative measurement of mRNA expression. For the isolation of mRNA, cells were cultivated in 12-*well* plates. Upon reaching confluency, mRNA was isolated using the GenElute-Mammalian Total RNA Miniprep Kit (Sigma, Taufkirchen, Germany). All steps are done according to manufacturer's recommendation. Prior to PCR analysis, cDNA was synthetized from the isolated RNA of the cell lines using the M-MLV Reverse Transcriptase Kit (200 u/µl, Invitrogen, Thermo Fisher Scientific, Waltham, MA, USA) according to manufacturer's recommendation. At the end of this process, cDNA was diluted in distilled water (1:20, v/v) and kept in freezer at -20 °C until use. RT-PCR analysis was performed using the ThermoPrime Taq DNA Polymerase (Thermo Fisher Scientific) Kit in a thermocycler under the following conditions: 1 cycle x 5 minutes at 95 °C (denaturation) followed by 35 cycles consisting of 30 seconds at 95 °C (denaturation), 30 seconds at 60 °C (annealing) and 1 minute at 72 °C (elongation) and finally 10 minutes at 72°C (elongation). The primers used in these analyses are listed in supplementary Table S1. DNA products were separated by a gel electrophoresis (80-90 Volt for about 20-30 minutes) using a 1.5% agarose gel containing ethidiumbromide. Finally, the intercalated DNA was excited with ultraviolet light and a picture was taken using AlphaImager (Biozym Scientific, Oldendorf, Germany). Real-time PCR was performed using SYBR Green PCR Master Mix and the ABI PRISM 7900 Sequence

Detection System (Applied Biosystems, Darmstadt, Germany) (primer pairs see supplementary Table S1). Gene expression is indicated by the Ct values, which are reported in supplementary Table S2.

**Supplementary Table S1.** Primers used for PCR analysis of OCT and GAPDH expression in HEK293 cells.

| | **Sequences of sense (S) and antisense (AS) primers (5′-3′)** |
|---|---|
| **Transporter** | |
| hOCT1 | S: CAT CAT AAT CAT GTG TGT TGG CC<br>AS: CAA ACA AAA TGA GGG GCA AGG CTT |
| mOCT1 | S: GCT GTT GTC TCA GAA GAG AAC C<br>AS: GAT GAG GCC CTG GTA CAG |
| hOCT2 | S: TCA TGG CCA TTT CCC AAC C<br>AS: CAC CAG GAG CCC AAC TGT AT |
| mOCT2 | S: AAA TCG GTG CCA GTC TCT CTT C<br>AS: TGC ATG ATG AGG CCC TGG |
| hOCT3 | S: GAC AAG AGA AGC CCC CAA CCT GAT<br>AS: CAC TAA AGG AGA GCC AAA AAT GTC |
| mOCT3 | S: CTG CCC AGC TTT CTC TTC CTC<br>AS: CTG ACT TCT TCA TCT GTA ACT |
| hMATE1 | S: AAG CTG GAG CTG GAT GCA GTC<br>AS: CAG CAG AGG AGC AGG ACG AGC |
| GAPDH | S: CAA GCT CAT TTC CTG GTA TGA C<br>AS: GTG TGG TGG GGG ACT GAG TGT GG |

hOCT1-3: human organic cation transporter 1-3; mOCT1-3: mouse organic cation transporter 1-3; hMATE1: human Multidrug and Toxin Extrusion Protein 1; GAPDH; glyceraldehyde-3-phosphate-dehydrogenase

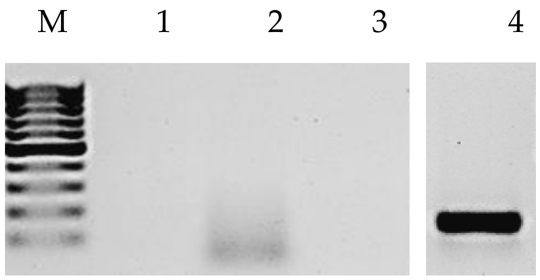


**Supplementary Figure S1**. mRNA expression of hOCT1-3 in WT-HEK293 cells: M) 100 bp marker. The other lanes show the signals for mRNA expression of 1) hOCT1, 2) hOCT2, 3) hOCT3, and 4) hGAPDH. For hOCT1 and hOCT3 no mRNA expression could be detected. In the lane 2 where mRNA expression of hOCT2 was tested, a tiny band below 100 bp dimension was visible, probably corresponding to primer dimers.

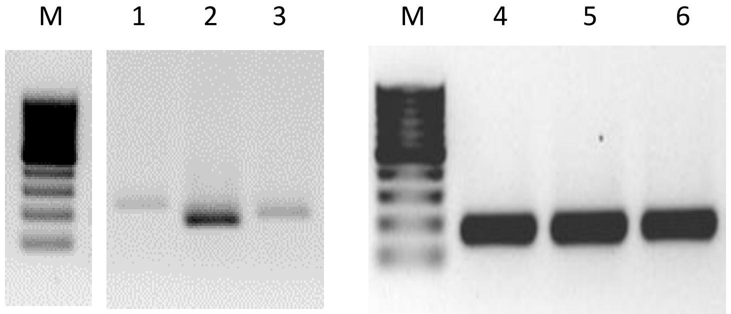


**Supplementary Figure S2**. mRNA expression of hOCT1-3 in HEK293 cells transfected with these transporters: the lanes M show the 100 bp marker. The lanes 1, 2, and 3 show the signals for hOCT1 mRNA in hOCT1-HEK293 cells, hOCT2 mRNA in hOCT2-HEK293 cells, and hOCT3 mRNA in hOCT3-HEK293 cells, respectively. The lanes 4-6 show the signals for the respective hGAPDH-mRNA content as a loading control.

**Supplementary Table S2.** Ct values (mean ± SEM) obtained by real-time PCR analysis of WT-HEK293 cells and of HEK cells stably transfected with hOCT1, hOCT2, or hOCT3.

| | **Ct values (mean ± SEM) of GAPDH- and hOCT1-3 mRNA expression in HEK293 cells transfected (hOCT1-3-HEK293) or not (WT-HEK293) with OCT. N indicates the number of independent experiments** | | | |
|---|---|---|---|---|
| **Cell line** | **GAPDH** | **hOCT1** | **hOCT2** | **hOCT3** |
| WT-HEK293 | 15.3 ± 0.3<br>N = 8 | 31.4 ± 0.2<br>N = 8 | 31.7 ± 0.7<br>N = 8 | 31.0 ± 0.1<br>N = 8 |
| hOCT1-HEK293 | 15.3 ± 0.2<br>N = 8 | 22.4 ± 0.3<br>N = 8 | n.d. | n.d. |
| hOCT2-HEK293 | 13.2 ± 0.7<br>N = 8 | n.d. | 16.2 ± 0.3<br>N = 8 | n.d. |
| hOCT3-HEK293 | 15.6 ± 0.2<br>N = 8 | n.d. | n.d. | 22.1 ± 1.2<br>N = 8 |

[1] n.d. = in this case the Ct value was not determined.

Results in Supplementary Figure S1 suggest no expression of hOCT1-3 in WT-HEK293 cells. These results were confirmed by real-time PCR analysis, where the Ct values for hOCT1-3 mRNA expression were above 30. Conversely, HEK293 cells transfected with hOCT1-3 confirm to have a much higher OCT-mRNA expression, as evident from the presence of well visible bands for transporter mRNA in Supplementary Figure S2, corresponding to the expected product length of amplification reaction, and from the lower Ct values obtained in the real-time PCT analysis, compared with what measured in WT-HEK293 cells (Supplementary Table 2).

Finally, with the help of semi-quantitative RT-PCR analysis we confirmed the overexpression of murine OCT in HEK293 cells transfected with mOCT1-3, as shown in Supplementary Figure S3.

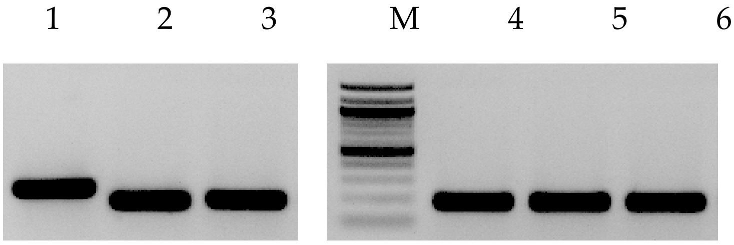


**Supplementary Figure S3**. mRNA expression of mOCT1-3 in HEK293 cells transfected with the murine transporter subtypes 1) mOCT1, 2) mOCT2, or 3) mOCT3. M is the 100 bp marker lane and 4), 5), and 6) the respective hGAPDH-mRNA content as a loading control.

Characterization of OCT expression by immunofluorescence analysis of the cells used in this study

For immunofluorescence analysis of transporter expression, cells (hOCT1-, hOCT2-, hOCT3-, mOCT1-, mOCT2-, and mOCT3-HEK293 cells) on cover slips were fixed at room temperature in 4% paraformaldehyde for 10 min. After fixation, cells were washed three times with phosphate-buffered saline (PBS) and incubated with 0.1% Triton X-100 for 3 min. After extensive washing with PBS, unspecific binding sites were blocked by overnight incubation at 4°C with 1% v/v gelatine (cold fish skin, Sigma). The cells were then incubated 60 min at room temperature with primary OCT antibodies (antibody against hOCT1 was from Santa Cruz

Biotechnology, Dallas, TX, USA; antibody against hOCT2 was a gift from Prof. Koepsell; antibody against hOCT3 and mOCT3 was from Abcam, Cambridge, UK; antibodies against mOCT1-2 were a gift from Prof. Koepsell) diluted 1:100. After three washing steps in PBS, the secondary antibody (goat-anti-mouse Alexa flour 488, Invitrogen) at a 1:1000 dilution was incubated for 60 min followed by five more washing steps in PBS. Negative control labelling was performed by omitting incubation with the primary antibody. Finally, the cells were covered with Crystal mount (Sigma). Fluorescence photographs were taken with an Axiocam camera mounted on Axiovert 100 microscope (Carl Zeiss, Göttingen, Germany) using Axiovision software. Pictures of transporter (supplementary Figures S4a and S5a) and negative control labelling (supplementary Figures S4b and S5b) were taken using identical illumination conditions.

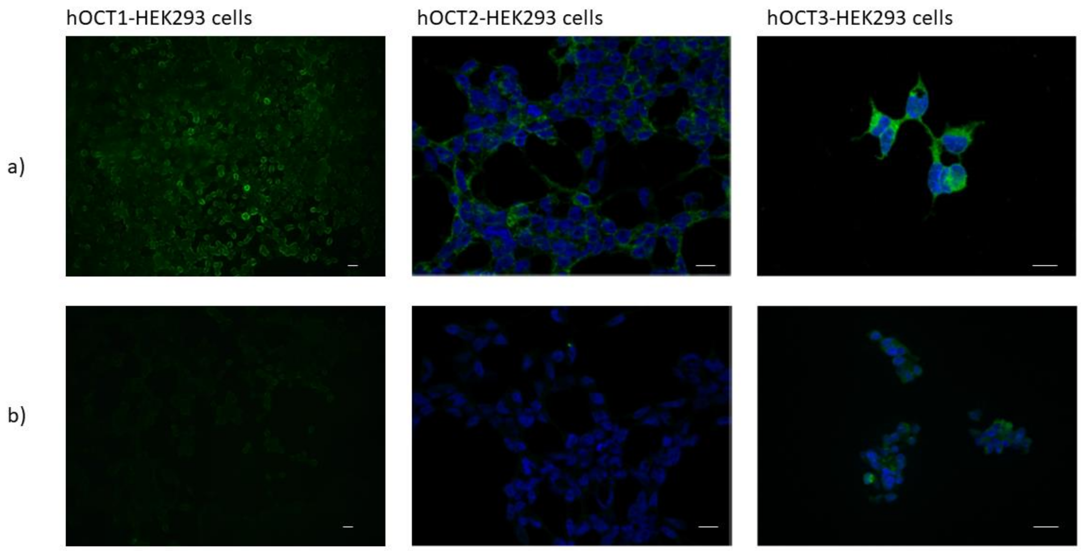


**Supplementary Figure S4**. Immunofluorescence analysis of transporter expression in hOCT1-3-HEK293 cells. The signal deriving from antibody labeling is shown in green. Nuclei are stained with 4,6-diamidino-2-phenylindole (DAPI, blue), except in the experiments with hOCT1-HEK293 cells, where nuclei were not labeled. The pictures in the line above (a) show the antibody labeling for the respective transporter, those in the line below (b) represents the labeling obtained in the absence of the primary antibody as a negative control experiment. The white bar shows a length of 10 μm.

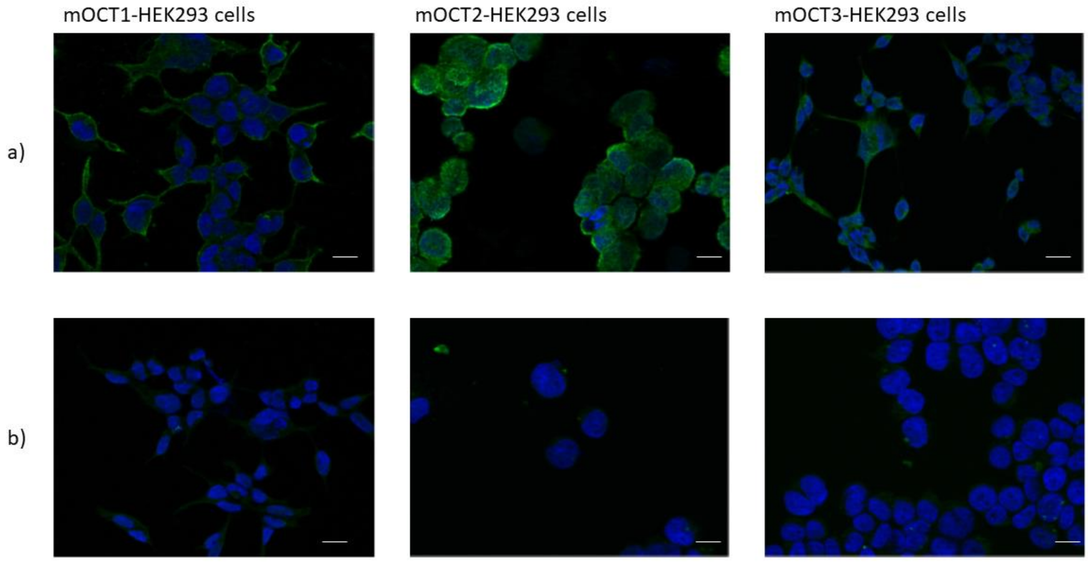


**Supplementary Figure S5**. Immunofluorescence analysis of transporter expression in mOCT1-3-HEK293 cells. The signal deriving from antibody labeling is shown in green. Nuclei are stained with 4,6-diamidino-2-phenylindole (DAPI, blue). The pictures in the line above (a) show the antibody labeling for the respective transporter, those in the line below (b) represents the labeling obtained in the absence of the primary antibody as a negative control experiment. The white bar shows a length of 10 µm.

We quantified the overexpression of hMATE relative to GAPDH in HEK cells by real-time PCR analysis (Supplementary Figure S6).

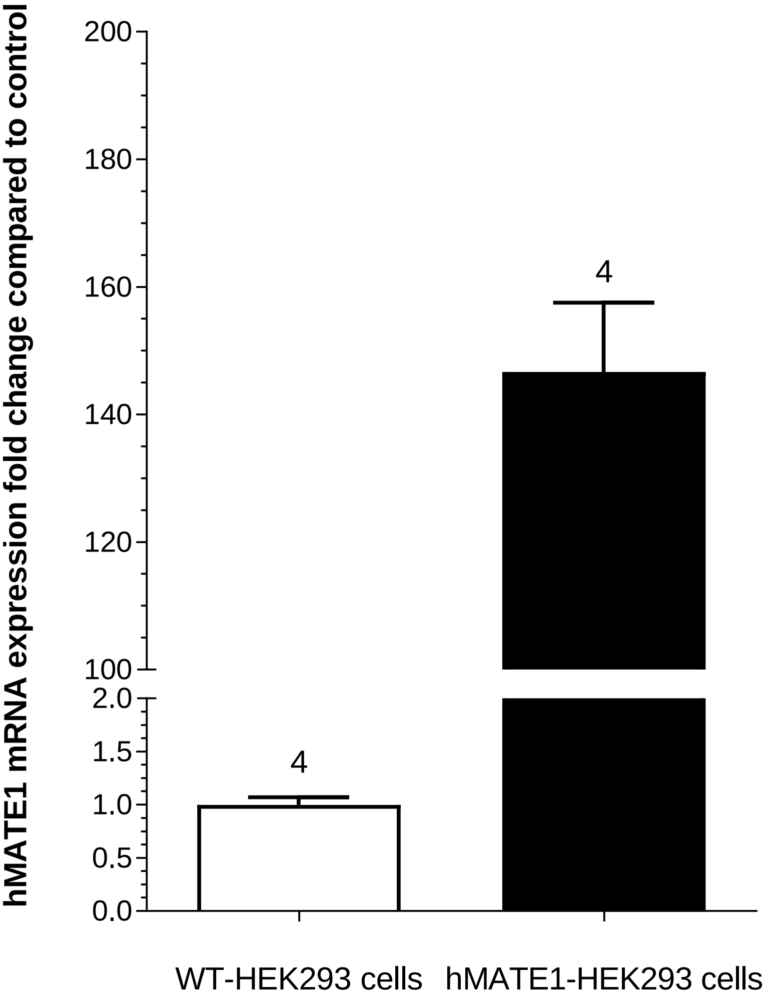


**Supplementary Figure S6.** Effects of hMATE1 transfection in HEK293 on hMATE1 mRNA expression compared with WT-HEK293 cells. Results are expressed as mean ± SEM, the number of experiments is indicated on the top of each column.

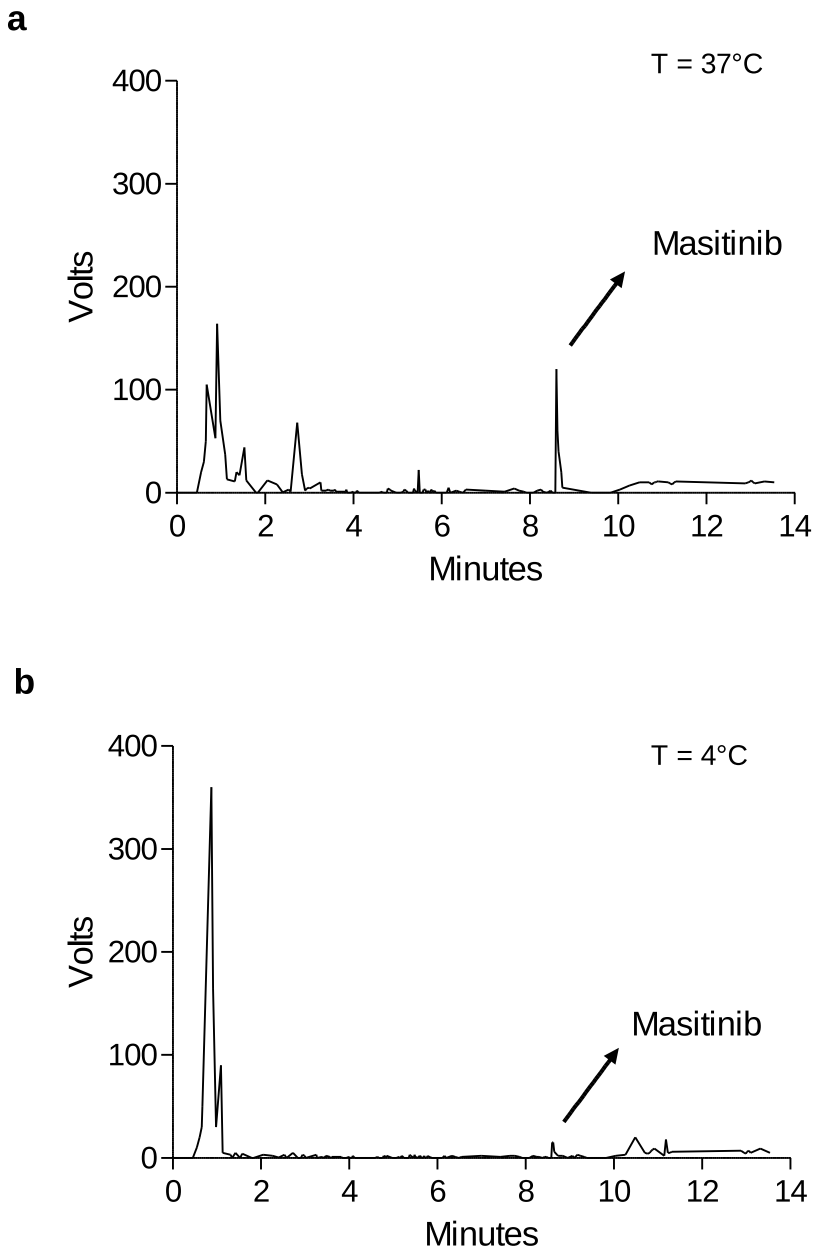


**Supplementary Figure S7.** Example of chromatograms for Masitinib detection in lysates from hOCT2-HEK293 cells after 10 minutes incubation with 10 μM Masitinib at 37 °C (panel a) and also under inhibition of metabolic processes at 4 °C (panel b). The arrows indicate the peak corresponding to Masitinib.
